# Regulation of cell-non-autonomous proteostasis in metazoans

**DOI:** 10.1042/EBC20160006

**Published:** 2016-10-15

**Authors:** Daniel O'Brien, Patricija van Oosten-Hawle

**Affiliations:** ^1^ School of Molecular and Cell Biology and Astbury Centre for Structural Molecular Biology, University of Leeds, Leeds LS2 9JT, U.K.

**Keywords:** cancer, cell-non-autonomous, heat-shock response, protein-misfolding diseases, proteostasis, stress responses, systemic proteostasis, transcellular chaperone signalling, unfolded protein response

## Abstract

Cells have developed robust adaptation mechanisms to survive environmental conditions that challenge the integrity of their proteome and ensure cellular viability. These are stress signalling pathways that integrate extracellular signals with the ability to detect and efficiently respond to protein-folding perturbations within the cell. Within the context of an organism, the cell-autonomous effects of these signalling mechanisms are superimposed by cell-non-autonomous stress signalling pathways that allow co-ordination of stress responses across tissues. These transcellular stress signalling pathways orchestrate and maintain the cellular proteome at an organismal level. This article focuses on mechanisms in both invertebrate and vertebrate organisms that activate stress responses in a cell-non-autonomous manner. We discuss emerging insights and provide specific examples on how components of the cell-non-autonomous proteostasis network are used in cancer and protein-folding diseases to drive disease progression across tissues.

## Introduction

The innate ability to cope with ever-changing environments and maintain the stability of the proteome is critical for organismal function and survival of all living systems. Organisms have developed an intricate network of components that maintain and re-establish protein homeostasis (proteostasis) in the face of proteotoxic stress in each cell type [1]. Components of the PN (proteostasis network) include molecular chaperones and their regulators, such as stress transcription factors and components of the proteolytic degradation machinery [2]. In eukaryotic cells, specific surveillance mechanisms such as stress signalling pathways have evolved to detect and respond to proteotoxic stress that occurs in the different cellular compartments. These stress signalling pathways synergize the co-ordination of the PN to maintain the stability of the proteome. This includes the cytosolic HSR (heat-shock response) [3] and organelle-specific stress responses such as the UPR (unfolded protein response) of the ER (endoplasmic reticulum) (UPR^ER^) and mitochondria (UPR^mito^) [4].

In addition to stress signalling responses that act locally and within the cell, the different cellular entities within a multicellular organism require intercellular signalling of stress responses between target tissues. Thus the HSR, the UPR^ER^ and the UPR^mito^ can be activated in a cell-non-autonomous manner by intercellular communication that allows organismal surveillance and regulation of proteostasis [5–7]. With the appearance of multicellularity, organismal proteostasis is augmented by forkhead transcription factors FOXO (forkhead box O)/DAF-16 and FOXA (forkhead box A)/PHA-4, which are also involved in the regulation of proteostasis signalling routes, such as transcellular chaperone signalling and FOXO-to-FOXO signalling [8,9].

Although it is clear that the PN is regulated in a co-ordinated and cell-non-autonomous fashion in metazoans, we are only beginning to understand the different layers of control that orchestrate the components of the PN at the systemic level. How is stress relayed between different cell types and organs? Which circulatory factors such as hormones and secreted peptides are utilized by the organismal PN to regulate and initiate different types of stress responses and at different levels of intensity?

In this article, we provide an overview of conserved cell-non-autonomous stress response mechanisms that maintain organismal proteostasis in invertebrates and vertebrates. We highlight emerging views indicating how organismal regulation of proteostasis is subverted by diseases such as cancer and protein misfolding diseases to successfully propagate the disease state across tissues. These examples may provide important clues about the identity of intercellular proteostasis signals and mechanisms that communicate proteostasis between different organs.

## Stress signalling pathways and their cell-non-autonomous regulation in metazoans

Within a multicellular context, cellular activation of stress signalling is superimposed by non-autonomous signalling responses by which the stressed tissue activates cytoprotective stress responses in different tissues and organs to facilitate survival of the organism as a whole. Stress signalling responses need to be co-ordinated throughout development and ageing, as unwarranted activation could be disadvantageous for the survival of the organism, if it were to perturb the complex interactions between tissues.

In this section, we address how the classical compartment-specific stress responses that traditionally have been known to function in a cell-autonomous manner, i.e. the cytosolic HSR, the UPR^ER^ and the UPR^mito^, can be activated in a cell-non-autonomous manner to allow for intercellular communication.

### Cell-autonomous and systemic regulation of the HSF1-mediated HSR

**Figure 1:**
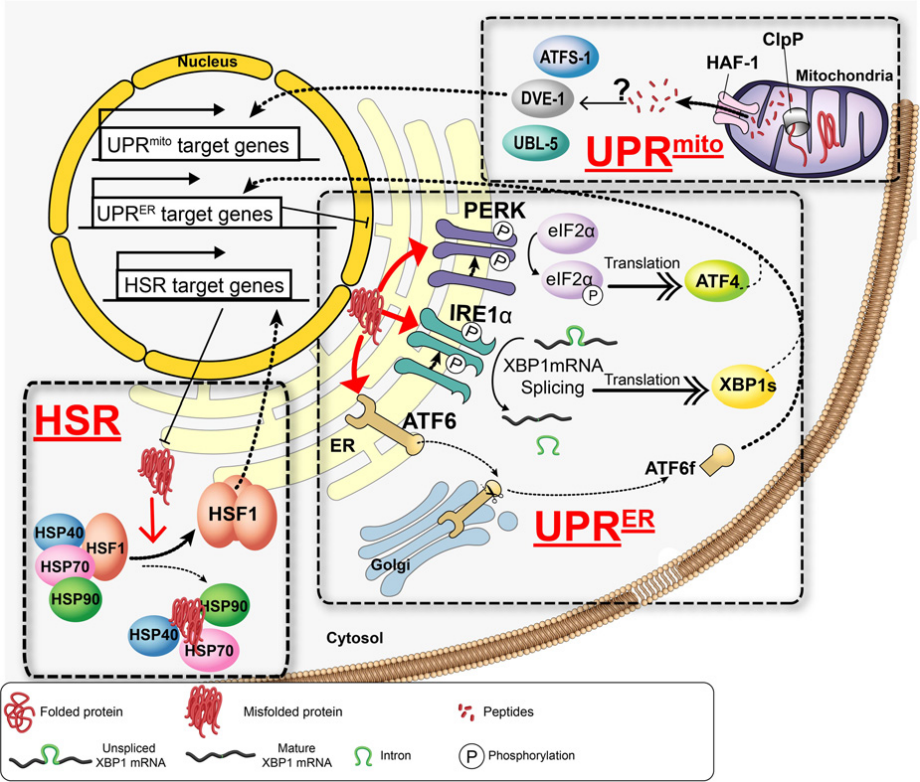
Stress signalling pathways regulating cellular proteostasis, including the HSR, the UPR^ER^ and the UPR^mito^ These stress response pathways can be triggered by environmental challenges which lead to protein folding stress and the accumulation of misfolded proteins. The HSR is regulated by HSF1. Proteotoxic stress results in the appearance of misfolded proteins in the cytosol, which releases HSF1 from its inhibitory interactions with HSP90, HSP70 and HSP40. This induces trimerization of HSF1 and translocation into the nucleus where it regulates expression of heat-shock genes. The UPR^ER^ is triggered by protein folding stress in the ER lumen, which activates transmembrane protein IRE1, PERK or ATF6, comprising the three branches of the UPR^ER^. Activation of IRE1 leads to splicing of *XBP-1* mRNA in the cytosol and translation of *XBP-1s*, the active transcription factor form which regulates expression of UPR-specific proteostasis components. The UPR^mito^ is activated upon protein misfolding in the mitochondrial matrix, which leads to cleavage of misfolded peptides by ClpP and subsequent transport into the cytosol by HAF-1. This results in formation of a transcriptionally active complex consisting of ATFS-1, DVE-1 and UBL-5 which induces transcription of mitochondria-specific chaperones.

The HSR is known as a cell-autonomous defence mechanism against thermal stress that is ancient and inherently displayed in bacterial cells as well as multicellular organisms of diverse taxa. In eukaryotes, the master regulator of the HSR is the highly conserved HSF1 (heat-shock transcription factor 1) [3] (Figure 1). When a cell is challenged by heat stress, HSF1 is converted from its inactive monomeric form that is sequestered by a multi-chaperone complex including HSP70 (where HSP is heat-shock protein) and HSP90 into a post-translationally modified trimeric form with DNA-binding capability [3]. In order to improve conditions for protein folding, activated HSF1 binds to a consensus sequence in the promoter of HSPs known as the HSE (heat-shock element) and leads to the rapid transcription of HSPs, such as the chaperone protein HSP70 [10]. After the imposed cellular stress has subsided, HSF1 activity is attenuated through a host of post-translational modifications including phosphorylation, SUMOylation and acetylation [3].

**Figure 2:**
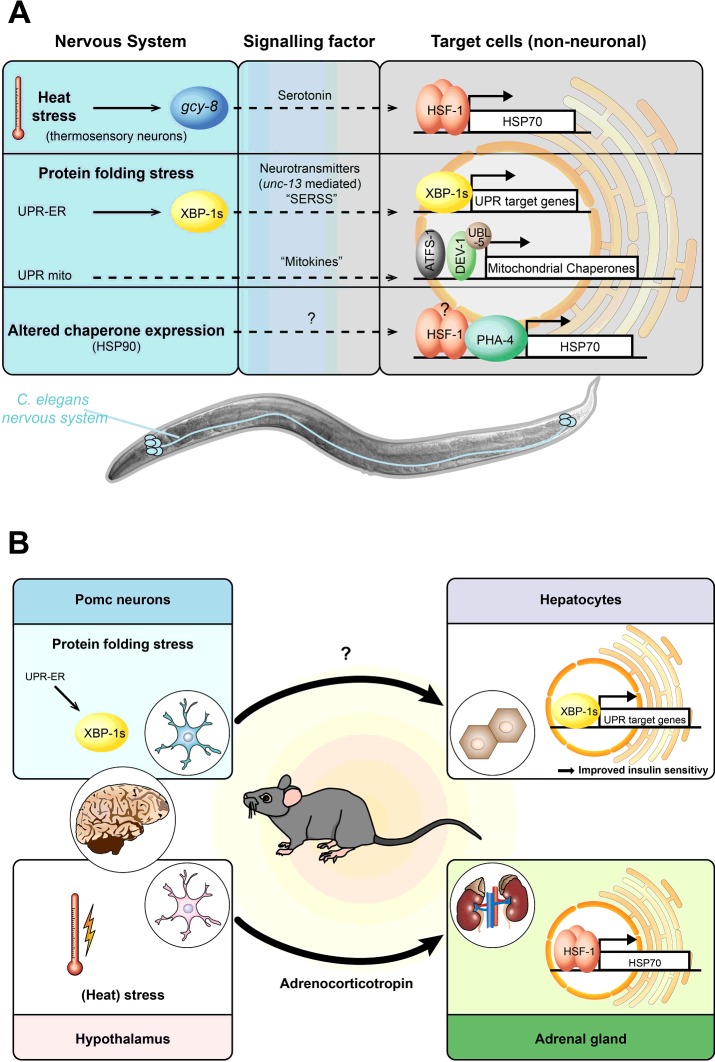
Stress signalling pathways regulating organismal proteostasis (**A**) *XBP-1s* cell-non-autonomous stress signalling in *C. elegans*. The HSR, the UPR^ER^ and the UPR^mito^ are regulated by the nervous system. The AFD neuron-directed cell-non-autonomous HSR in *C. elegans* requires activation of a signalling pathway that depends on the neuronally expressed guanylate cyclase 8 (*gcy-8*). Transcellular chaperone signalling can also occur independent of neuronal control when activated in non-neuronal tissues, such as muscle or gut [9]. (**B**) Cell-non-autonomous stress signalling in mammals. Expression of transcriptionally active *XBP-1s* in the POMC neurons of mice triggers cell-non-autonomous XBP-1 splicing and activation of the UPR in hepatocytes (liver), resulting in improved glucose utilization and insulin sensitivity. Rats subjected to restraint stress produce stress hormones along the hypothalamus–pituitary–adrenal axis (such as adrenocorticotropin) which activates HSF1 in the adrenal gland of the kidney and hence up-regulates the expression of HSP70.

In metazoans, the cell-non-autonomous activation of the HSR requires the integration of sensory and behavioural components to achieve optimal survival and adaptation. This integration is primarily controlled by the nervous system. In *Caenorhabditis elegans*, two thermosensory AFD neurons control thermotactic behaviour that direct the worm to the optimal ambient growth temperature [11]. On the molecular level, thermal stress propagates a signal from the AFD neurons via a *gcy-8* (guanylate cyclase 8)-dependent signalling cascade to peripheral tissues that stimulates HSF1 activity and expression of HSPs in muscle cells, the gut and reproductive organs [5,12] (Figure 2A). Recently, the transcellular signal that couples thermosensory neural activity with activation of cell-non-autonomous HSF1-mediated chaperone expression has been identified as serotonin. Remarkably, serotonergic signalling can also be activated by optogenetic excitation of the AFD neurons to induce HSF1 activity in distal tissues, even in the absence of damaging environmental conditions [12].

The importance of the neuronal integration of both behavioural and molecular responses is furthermore demonstrated by the fact that animals expressing mutated *HSF1* also show a defect in thermotactic behaviour. This behavioural defect can be suppressed by cell-specific expression of HSF1 in peripheral tissues such as muscle or gut cells [13]. A feedback mechanism from the muscle or gut, mediated by the oestrogen biosynthetic pathway, activates the neuronally expressed oestradiol nuclear receptor NHR-69 and thereby suppresses the defective behaviour of the worms [13].

Despite the study by Prahlad et al. [5] being the first to recognize that proteostasis-related stress signalling of the HSF1-mediated HSR acts in a cell-non-autonomous manner, similar observations in rodents reach back far earlier. Rats exposed to restraint stress release adrenocorticotropin from the pituitary gland in the hypothalamus, which consequently activates HSF1 in the adrenal gland and up-regulates HSP70 expression [14] (Figure 2B). This demonstrates that neuronal control of HSF1 activation in peripheral tissues also exists in mammals.

### Cell-autonomous and systemic regulation of the unfolded protein response of the ER (UPR^ER^)

The UPR^ER^ is activated upon the accrual of unfolded proteins in the lumen of the ER of eukaryotic cells. The UPR^ER^ is composed of three signalling branches controlled by the stress sensors IRE1 (inositol-requiring enzyme 1), PERK [PKR (dsRNA-dependent protein kinase)-like endoplasmic reticulum kinase] and ATF6 (activating transcription factor 6) (Figure 1) which are all localized in the ER membrane in order to facilitate transduction of signalling between the ER and the nucleus [4]. The IRE1 signalling pathway is conserved from yeast to mammals and so is the most highly studied of the three pathways [4]. Upon recognition of an unfolded protein, IRE1 oligomerizes and undergoes trans-autophosphorylation, which induces its endoribonucleic activity that allows cleaving of XBP-1 (X-box-binding protein 1) mRNA transcripts to their functional form, *XBP-1s*. *XBP-1s*, once trimmed and translated, enters the cell nucleus and directs activation of ER genes, such as the ER HSP70 chaperone GRP78 (glucose-regulated protein of 78 kDa)/BiP (immunoglobulin heavy-chain-binding protein), to combat the stress [4].

The UPR^ER^, like the HSR, is regulated in a cell-non-autonomous manner, with the nervous system again playing an apparent predominant role in the activation of the response across tissues. In *C. elegans*, XBP-1 is controlled by OCTR-1, an octopamine G-protein-coupled catecholamine receptor that is expressed in sensory neurons. This OCTR-1-mediated regulation of the canonical UPR^ER^ is switched on as *C. elegans* enters adulthood and is absent during embryonic and post-embryonic development of the worm. A constitutively active XBP-1 is required during developmental stages to meet the higher demands for protein synthesis and folding [15]. Interestingly, expression of the spliced and active form of the transcription factor XBP-1, *XBP-1s*, in *C. elegans* neurons activates XBP-1 in the intestine and induces expression of GRP78/BiP in this target tissue [7] (Figure 2A). This leads to increased stress resistance and lifespan of the worm. The transcellular neuronal signal that activates XBP-1 in the periphery is currently unknown. Its action, however, depends on functional neuronal vesicle release, which is mediated by the protein UNC-13 [7]. This result suggests that there is a SERSS (secreted ER stress signal), which allows signalling between neurons and other tissue types to promote ER stress resistance and longevity.

Consistent with the specialized function of the ER for the secretion of proteins required for lipid biosynthesis and membrane remodelling, the ER and the UPR^ER^ are involved in metabolic energy regulation, which has implications for diseases such as obesity and diabetes [16]. Within that context, it was recently discovered that transcellular UPR^ER^ activation also exists in mammals and is not a process restricted to invertebrates [17]. Expression of *XBP-1s* in the POMC (pro-opiomelanocortin) neurons in mice, which respond to insulin and leptin, led to XBP-1 splicing and hence activation of the UPR^ER^ in the liver (Figure 2B). This resulted in improved insulin sensitivity and increased energy expenditure and so protected mice from diet-induced obesity [17].

### Cell-autonomous and systemic regulation of the unfolded protein response of the mitochondria (UPR^mito^)

The UPR^mito^ also relies on the up-regulation of specialized chaperone proteins such as HSP60 and mtHSP70 (mitochondrial HSP70) to promote a functional protein-folding environment in the organelle. The mitochondrial proteostasis capacity can be disturbed by increased levels of ROS (reactive oxygen species), which are generated from the ETC (electron transport chain) and directly perturb protein folding and structure. Mutations in ETC components can also stress the mitochondrial protein-folding environment by impairing the assembly of the individual ETC complexes [18].

In *C. elegans*, excess unfolded protein in the mitochondrial matrix is cleaved into shorter peptide fragments by the ATP-dependent protease ClpP. The peptides are then pumped out of the matrix by HAF-1, the half-transporter 1 ATP-binding cassette transporter protein, which spans the mitochondrial inner membrane. It is thought that the translocated peptides can then freely cross the outer membrane and into the cytosol. This activates ATFS-1 (activating transcription factor associated with stress 1) which accumulates in the nucleus. The HAF-1-mediated efflux of peptides into the cytosol also leads to the accumulation of the ubiquitin-like protein UBL-5, which forms a transcriptionally active complex with DVE-1 in the nucleus (Figure 1). ATFS-1 and DVE-1/UBL-5 then co-operatively induce the transcription of mitochondrial chaperones [18].

Mutations that cause ETC dysfunction or reduced expression of ETC components are known to extend lifespan significantly in *C. elegans*, fruitflies and mice and is achieved, at least in part, by UPR^mito^ activation at a systemic level. In *C. elegans*, [6] the cell-non-autonomous activation of the UPR^mito^ appears to be controlled again by the nervous system [6]. Here, reduced neuronal expression of an ETC subunit component, *cco-1*, activates the expression of mtHSP70 in the intestine and is sufficient to extend lifespan of the worm [6]. Transcellular signalling of mitochondrial stress in other invertebrate models as well as vertebrates can, however, also originate from muscle cells. For example, mitochondrial damage in *Drosophila* flight muscles activates the expression of ImpL2, an insulin-antagonizing molecule, which reduces insulin signalling in muscle cells and in distal tissues [19]. Perturbation of mitochondrial function in muscle tissue of mice leads to secretion of FGF-21 (fibroblast growth factor 21) from muscle cells into the plasma [20]. This results in increased insulin sensitivity and activates a compensatory stress response involving the PERK branch of the UPR^ER^ [21].

In addition to the cell-non-autonomous co-ordination of the major stress-responsive transcription factors (HSF1, XBP-1 and ATFS-1), the metazoan proteostasis network also comprises specific forkhead family transcription factors such as FOXO/DAF-16 and FOXA/PHA-4 that mediate expression of proteostasis components between tissues. FOXA/PHA-4 is involved in a process called transcellular chaperone signalling, which allows the regulation of chaperone expression through cross-tissue communication. FOXO/DAF-16 is, in addition to its major role in aging and youthfulness, involved in FOXO-to-FOXO signalling. Both of these transcellular signalling processes can be regulated independently of neuronal control.

### Transcellular chaperone signalling

Transcellular chaperone signalling can be activated from any tissue, through changing tissue-specific expression levels of the major chaperone HSP90. This activates a compensatory chaperone response in different target tissues, which depends on the conserved forkhead transcription factor FOXA/PHA-4. PHA-4, in addition to HSF1, recognizes a consensus sequence in the promoter of the *hsp90* gene and hence regulates *hsp90* expression in *C. elegans* (Figure 2B) [9].

Whereas transcellular chaperone signalling can be activated from the neurons to induce cell-non-autonomous adjustment of *hsp90* and *hsp70* expression in peripheral tissues, it can also be triggered from gut or muscle cells by altered expression of *hsp90*. In this instance, cell-non-autonomous regulation of *hsp90* or *hsp70* expression is independent of neuronal control [9]. Because HSP90 is part of the negative-feedback mechanism that represses HSF1 activity after heat shock, this can, however, be deleterious to the organismal survival during heat stress. Conversely, tissue-specific *hsp90* knockdown in the neurons or intestine causes a compensatory and PHA-4-dependent up-regulation of *hsp70* in distal target tissues and enhances stress resistance and organismal survival. This indicates that it is the balance of chaperone proteins at an organismal level that determines intercellular signalling, rather than abundance or scarcity [9]. Importantly, this inter-tissue regulation of cytoprotective chaperone expression through transcellular chaperone signalling has the potential to improve proteostasis in tissues affected by protein misfolding. For instance, transcellular activation of *hsp90* expression from neurons, was shown to suppress misfolding of metastable myosin expressed specifically in muscle tissue [9].

### Insulin and FOXO-to-FOXO signalling

Insulin and ILS (insulin-like signalling) are one of the most studied mechanisms that can modify aging in a range of organisms [22–24]. It functions through regulating activity of FOXO that is conserved from invertebrates to mammals. Regulation of lifespan extension and maintenance of proteostasis are inherently linked through this signalling pathway [25,26].

FOXO transcription factors are themselves implicated in transcellular proteostasis signalling activities to enhance and maintain cell-non-autonomous proteostasis. In *C. elegans*, expression of FOXO/DAF-16 in the gut induces target gene expression in muscle cells through a process known as FOXO-to-FOXO(-) signalling that prevents age-related deterioration and protects against the toxic effects of Aβ (amyloid β-peptide)-associated protein misfolding in the muscle [8]. Likewise, increased expression of the FOXO target 4E-BP (eukaryotic initiation factor 4E-binding protein) in *Drosophila* muscle cells extends lifespan and enhances proteostasis in the entire animal, through a mechanism that changes feeding behaviour and hence reduces insulin signalling [27]. These FOXO-dependent cell-non-autonomous effects on organismal proteostasis are universal and conserved. For example, tissue-specific knockout of the IGF-1 receptor in adipose tissue of mice, which controls FOXO activity, is sufficient to extend lifespan [28]. Moreover, reduced IGF-1 expression delays proteotoxicity associated with protein misfolding diseases, such as shown for a murine Alzheimer's disease model [29].

## The dark side: cell-non-autonomous proteostasis mechanisms hijacked by diseases

Although the organismal proteostasis network has developed to fine-tune and adjust the stress response output in different cell types simultaneously, it can also be subverted to facilitate disease progression across tissues. Expression of aggregated disease proteins, even when localized to a specific tissue or cell type does have a global impact on different aspects of proteostasis regulation. Huntington's disease-associated polyQ (polyglutamine) expansion proteins that are expressed in the *C. elegans* bodywall muscle and intestinal tissue inhibit neuronal cues that otherwise allow activation of HSF1 and the protective up-regulation of chaperone expression [30]. Such localized expression of aggregated proteins also have a widespread impact on ribosomal translation rates in a cell-non-autonomous manner, as demonstrated for muscle-specific expression of Aβ and polyQ in *C. elegans* [31].

Propagation of protein aggregates throughout the nervous system by cell-non-autonomous processes plays a crucial and significant role in the pathology of many neurodegenerative diseases, including ALS (amyotrophic lateral sclerosis), Huntington's disease and Alzheimer's disease [32]. An increasing body of studies now provide evidence that spreading of disease proteins within the brain of invertebrates and mammals can occur via multiple mechanisms that includes exosomes and tunnelling nanotubes [33], as well as through hijacking neuronal exocytosis and endocytosis mechanisms [34,35]. Along the same lines, prion proteins expressed in a specific tissue or cell are known to hijack intercellular trafficking pathways that allows intercellular spreading and impairs the cell-non-autonomous protein-folding environment, in *C. elegans* as well as in mammalian cells [36,37].

These findings are underscored further by emerging insights from cancer cell studies. Both the HSF1- and UPR^ER^-mediated stress signalling pathways are utilized by cancer cells to cell-non-autonomously advance neoplastic growth [38,39]. Although it is well known that cancer cells relay micro-environmental stresses to clinically normal cells, the recent realization that HSF1 activity and UPR signalling can be regulated in a cell-non-autonomous manner gives these observations in tumour biology particular importance, as they might provide clues for intercellular signalling mechanisms that regulate proteostasis in an organism.

### Cancer and cell-non-autonomous regulation of HSF1

**Figure 3:**
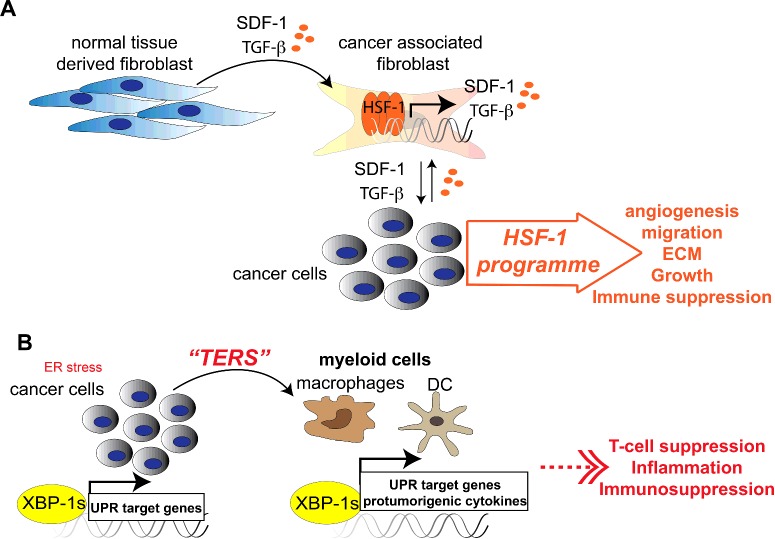
Cell-non-autonomous regulation of stress signalling pathways in cancer cells (**A**) Cell-non-autonomous activation of HSF1 in cancer cells. Cancer-associated fibroblasts within the tumour microenvironment express and release SDF-1 and TGF-β in an HSF1-dependent manner. This activates an HSF1 transcriptional programme in tumour cells that further promotes tumour cell growth. (**B**) TERS in cancer. Activation of the UPR^ER^ in cancer cells leads to the secretion of factors that activate the UPR in receiving myeloid cells (macrophages and DCs).

The ability of cancer cells to proliferate and induce metastasis depends on their ability to reprogramme clinically healthy stromal cells, which are naturally present in the tumour microenvironment, such as cancer-associated fibroblasts. Activated stroma cells in this microenvironment express and secrete TGF-β (transforming growth factor β) and SDF-1 (stromal D factor 1), which allows for intercellular communication with cancer cells. Quite strikingly, the expression of these secretory factors is achieved in an HSF1-dependent manner as SDF-1 harbours an HSE bound by HSF1 [38]. Following secretion of SDF-1 and TGF-β, HSF1 is subsequently activated in neighbouring cancer cells where it drives the expression of genes that facilitate adhesion, migration and extracellular matrix organization, i.e. factors that further promote tumour growth [38] (Figure 3A). These diverse cancer-related HSF1 transcriptional programmes were uncovered relatively recently by the Lindquist laboratory [38,40] and notably demonstrate the range of HSF1 target genes, which not only include the traditionally known heat-shock genes, but a remarkably large fraction of target genes required for transcellular communication between stroma and cancer cells [38,40].

### Cancer and cell-non-autonomous regulation of the UPR^ER^

Another observation demonstrating the subversion of intercellular stress signalling to benefit tumorigenic growth comes from the UPR^ER^. Tumour cells undergoing ER stress have the capability to transmit and activate the UPR^ER^ in myeloid cells, such as macrophages and DCs (dendritic cells) [39,41]. This not only induces the UPR^ER^ in these cell types, but also modifies the transcriptional output to support cancer cell growth. Alongside an up-regulation of *XBP-1s* and induction of downstream effectors such as GRP78 in macrophages and dendritic cells, this also leads to secretion of pro-tumorigenic cytokines, including TGF-β. The identity of the signalling molecule secreted by tumour cells, termed TERS (transmissible ER stress), that induces the UPR^ER^ in receiving myeloid cells is, however, currently unknown (Figure 3B).

## Consequences and future developments

In this article, we have described how the traditional cellular stress response mechanisms are integrated in metazoans to allow inter-tissue communication during stress conditions. By now, it is clear that the cell-non-autonomous regulation of stress signalling pathways not only occurs in invertebrate model organisms, but is a process that is conserved in vertebrates as well.

Both understanding the cellular pathways involved in the spreading of disease, such as pathogenic proteins and propagation of neoplastic growth, and identification of signals that mediate the propagation of stress responses in a cell-non-autonomous manner will be a particularly crucial next step for the field. This is because transcellular stress signalling opens up unprecedented potential for novel therapeutic interventions. Recent examples from invertebrate models have shown that stimulating somatic cells in the absence of stress can improve the folding capacity of cells in the periphery that are affected by misfolded protein diseases [8,9,12]. Invertebrate models may be ever more important in the development of new therapeutic avenues, as they allow unbiased genome wide screens to identify the as yet enigmatic transcellular signal that induces cell-non-autonomous regulation of proteostasis.

Examples from diseases such as cancer show that it may be necessary to look beyond classical mechanisms of activation of stress responses to find the transcellular signal. Clearly, distinct stimuli can engage HSF1 and the UPR in the absence of molecular signatures that are otherwise protective. The answer may not be as simple as ‘one signal that regulates it all’, but an intricate network consisting of a variety of metabolites, secreted peptides and hormones that activate distinct components of the organismal proteostasis network.

## Summary

- Cellular proteostasis is regulated by a cohort of stress signalling pathways including the HSR, the UPR^ER^ and the UPR^mito^.
- Organismal proteostasis is regulated by cell-non-autonomous activation of these stress signalling pathways.
- Cell-non-autonomous stress signalling can be controlled by neuronal cues as shown for the HSR, UPR^ER^, and UPR^mito^, but can also be independent of the nervous system by transcellular chaperone signalling and FOXO-to-FOXO signalling.
- Cell-non-autonomous stress signalling pathways mediated by HSF1 and the UPR^ER^ are used by cancer cells to propagate tumour growth.

## Abbreviations:

Aβ: amyloid β-peptide
ATF6: activating transcription factor 6
ATFS-1: activating transcription factor associated with stress 1
BiP: immunoglobulin heavy-chain-binding protein
DC: dendritic cell
ER: endoplasmic reticulum
ETC: electron transport chain
FOXA: forkhead box A
FOXO: forkhead box O
GRP78: glucose-regulated protein of 78 kDa
HSE: heat-shock element
HSF1: heat-shock transcription factor 1
HSP: heat-shock protein
HSR: heat-shock response
IRE1: inositol-requiring enzyme 1
mtHSP70: mitochondrial HSP70
PERK: PKR (dsRNA-dependent protein kinase)-like endoplasmic reticulum kinase
PN: proteostasis network
polyQ: polyglutamine
POMC: pro-opiomelanocortin
SDF-1: stromal D factor 1
TERS: transmissible ER stress
TGF-β: transforming growth factor β
UPR: unfolded protein response
UPR^ER^: UPR of the ER
UPR^mito^: UPR of the mitochondria
XBP-1: X-box-binding protein 1
